# Machine learning classification of schizophrenia patients and healthy controls using diverse neuroanatomical markers and Ensemble methods

**DOI:** 10.1038/s41598-022-06651-4

**Published:** 2022-02-17

**Authors:** Geetha Soujanya Chilla, Ling Yun Yeow, Qian Hui Chew, Kang Sim, K. N. Bhanu Prakash

**Affiliations:** 1 Institute of Bioengineering and Bioimaging, Agency for Science, Technology and Research, Singapore, Singapore 138667; 2 Institute of Mental Health, Singapore, Singapore 539747

**Keywords:** Computational neuroscience, Image processing, Machine learning, Psychiatric disorders

## Abstract

Schizophrenia is a major psychiatric disorder that imposes enormous clinical burden on patients and their caregivers. Determining classification biomarkers can complement clinical measures and improve understanding of the neural basis underlying schizophrenia. Using neuroanatomical features, several machine learning based investigations have attempted to classify schizophrenia from healthy controls but the range of neuroanatomical measures employed have been limited in range to date. In this study, we sought to classify schizophrenia and healthy control cohorts using a diverse set of neuroanatomical measures (cortical and subcortical volumes, cortical areas and thickness, cortical mean curvature) and adopted Ensemble methods for better performance. Additionally, we correlated such neuroanatomical features with Quality of Life (QoL) assessment scores within the schizophrenia cohort. With Ensemble methods and diverse neuroanatomical measures, we achieved classification accuracies ranging from 83 to 87%, sensitivities and specificities varying between 90–98% and 65–70% respectively. In addition to lower QoL scores within schizophrenia cohort, significant correlations were found between specific neuroanatomical measures and psychological health, social relationship subscale domains of QoL. Our results suggest the utility of inclusion of subcortical and cortical measures and Ensemble methods to achieve better classification performance and their potential impact of parsing out neurobiological correlates of quality of life in schizophrenia.

## Introduction

Psychotic spectrum disorders such as schizophrenia affect individuals in multiple domains including cognitive domains, interpersonal relationships and daily psychosocial functioning^1^. Diagnosis of these disorders is carried out through detailed history taking, mental status examination, clinical examination and laboratory investigations whenever appropriate to rule out organic causes^2,3^. Apart from the use of clinical rating scales to assess the severity of psychopathology, there are increasing efforts in the identification of genetic, biochemical, or imaging biomarkers^4–7^ that could aid in diagnosis, treatment and prognosis of illnesses and their subtypes. Elucidation of such underlying biomarkers could complement extant clinical measures and provide information regarding neural substrates underlying illness status.

Of note, neuroimaging studies have revealed abnormalities involving structural and functional cerebral changes within schizophrenia involving cortical (such as frontal region), subcortical regions (such as hippocampus, thalamus) and network connectivity alterations^8–14^. Specifically, there is increasing interest in the employment of structural neuroimaging features to improve the diagnosis of schizophrenia using machine learning methods^15–17^. Guo et al. employed features from amygdaloid and hippocampal subregions to differentiate between healthy controls and schizophrenia patients^15^. They carried out feature selection using sequential backward elimination and utilized Support Vector Machine Classifier (SVC)/(SVM) from which they reported an accuracy of 81.75% with sensitivity of 84.21%. In another study^16^, authors Yassin et al. carried out classification on a dataset consisting of 64 schizophrenia patients and 106 healthy controls using subcortical volumes and cortical thickness features. Highest accuracies of 76.4% were achieved using subcortical volumes as features and a random forest classifier, 70.5% using cortical thickness as features and a decision tree analysis and 70.5% using both subcortical volumes and cortical thickness features and logistic regression as a classifier. Xiao et al. carried out classification on 163 first-episode drug-naïve schizophrenia patients and 163 healthy controls. Using cortical thickness and cortical surface area, they achieved accuracy and sensitivity in the range of 81–85% and 77–83% respectively^17^.

While there have been efforts to differentiate between patients with schizophrenia and healthy controls within subject cohorts, there are limited studies which employed a wider range of neuroanatomical measures for such classification. Even when more than one set of measures were used with machine learning algorithms, classification performance may not necessarily increase^16^. In this regard, Ensemble methods are multiple classifier systems where individual weak classifiers are combined to generate a more robust classification system. Outputs from multiple base learning algorithms are voted or stacked through an algorithm in training to generate an Ensemble classifier which can then classify new data. Based on extant data and possible benefit of Ensemble methods in strengthening classifiers, we hypothesize that employing a diverse set of neuroanatomical measures with Ensemble classification methods will improve classification performance. Hence, in a bid to improve on the accuracy and sensitivity of classification between schizophrenia and healthy controls, we employed a wider range of neuroanatomical features (cortical thickness, surface area, volume, mean curvature, subcortical volumes) with Ensemble methodology to improve overall performance of classification. We further correlated neuroimaging measures with quality of life measures to gain further insights into the relationship between neuroimaging measures and the functional status of our subjects.

## Methods

### Subject recruitment and study details

Patients with schizophrenia (*n* = 158) were recruited from Institute of Mental Health, Singapore. Confirmation of the diagnosis was made for all patients by psychiatrists based on information obtained from clinical history, existing medical records, interviews with significant others as well as administration of the Structured Clinical Interview for DSM-IV Disorders-Patient Version (SCID-P)^18^. There was no history of any significant neurological illness such as seizure disorder, head trauma or cerebrovascular accident for the patients. Healthy controls were recruited from the community by advertisements. Control subjects (*n* = 76) were free of any Axis I psychiatric disorder as determined by the SCID-Patient version (SCID-NP)^19^ and had no history of any major neurological, medical illnesses, substance abuse or psychotropic medication use. Written, informed consent was acquired from all the participants after a detailed explanation of the study procedures. The study protocol was approved by the Institutional Review Boards of both Institute of Mental Health and the National Neuroscience Institute, Singapore. All methods were performed in accordance with the relevant guidelines and regulations.

MR Imaging was carried out for patients and healthy controls on 3 T Philips Achieva scanner (Philips Medical Systems, Eindhoven, The Netherlands) using parallel imaging (SENSE). Axial T1 MPRAGE volumes were acquired with a matrix size of 256 × 256 and a resolution of 0.8984 × 0.8984 × 1 mm^3^, with at least 180 slices covering the brain.

**Figure 1:**
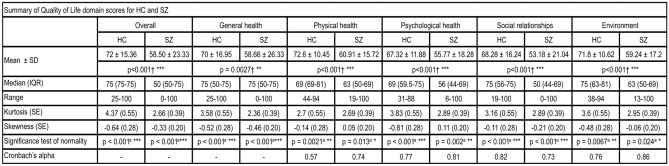
Summary of QoL items and domain scores prior to transformations—QoL scores are significantly higher in the healthy controls than patients with schizophrenia in all items. p-value (*p < 0.05**p < 0.01***p < 0.001) was calculated by ^†^Mann–Whitney U-test, and ^a^Shapiro–Wilk’s test.

Quality of Life (QoL) for subjects was assessed using the World Health Organization Quality of Life assessment—Brief Form (WHOQOL-BREF)^20^, which is a 26-item, 5-point self-rated questionnaire. It assesses subjective QoL in four domains, namely physical health (7-items), psychological (6-items), social relationships (3-items), and environment (8-items), with the 2 remaining items assessing overall perception of QoL and overall health satisfaction. After reverse-scoring for items 3, 4 and 26, raw scores within each domain were standardized to 0–100 range to obtain a domain score. A higher score indicates better subjective QoL. A summary of QoL for all domains and items in healthy controls and schizophrenia cohort is given in Fig. 1.

### Image processing

To extract neuroanatomical measures, image data has been converted to NIfTI format using dcm2nii^21^ given the retrospective nature of the data. Subcortical segmentation and cortical surface reconstruction has been carried out using Freesurfer 6.0.0 software^22–40^. In this reconstruction process, subcortical regions were segmented using Gaussian Classifier Atlas^41^ from which 55 subcortical features including sub-region volumes, white matter and non-white matter subcortical hypo-intensities were obtained. Cortical parcellation was carried out using Desikan–Killiany atlas^42^ through which 71 cortical volume features, 73 cortical surface area features, 71 cortical mean curvature features and 73 cortical thickness features were obtained. Reconstruction process on the entire dataset was performed using GNU parallel^43^ on a high performance computing platform at National Supercomputing Centre (NSCC), Singapore.

In addition to visual inspection of images, quality assurance of data has been carried out through histogram based and quantile–quantile plots using generated neuroanatomical features to ensure that no significant abnormalities were included in the analyzed dataset.

### Machine learning based classification

A total of 5 measures, namely cortical and subcortical volume, cortical surface area, cortical mean curvature and cortical thickness were obtained for classification of patients as mentioned in the previous section. In the first set of analyses, these measure sets were independently employed for classification and in the second set of analyses, all measures were merged or used in Ensemble for classification, as explained in detail in “Study design”. For all analyses, data was standardized, feature selection and train-test splitting were carried out before classifier selection and hyper-parameter tuning.

#### Standardization, feature selection and train-test split

All features were standardized to zero mean and unit variance using *StandardScaler* of Sklearn library. Feature selection was then carried out where most important features were selected using *SelectFromModel* of Sklearn library, using an SVC estimator. For training and test datasets generation, data was split in the ratio 70:30. Class proportions of 1:2 between healthy control cohort and schizophrenia cohort were maintained in training and test datasets and class balancing was employed with all estimators and classifiers wherever applicable. Final composition of the training and test dataset was 163 and 71 samples respectively.

#### Classifier selection and hyper-parameter tuning

To achieve best classification performance, initial classifier selection was done on its baseline performance and then was further optimized using best hyper-parameters. Multiple classifiers including k-Nearest Neighbors, Logistic regression, SVM classifiers (SVC with radial basis function kernel, Linear SVC, Nu-SVC), Decision trees, Random forests were tested for classifier selection process using F1 score and Area under Curve (AUC) as performance metrics. Once the base classifier was selected, tuning was carried out through exhaustive search over parameter space using *GridSearchCV*. A threefold cross-validation was done on training dataset during optimization and classification performance was evaluated on accuracy, AUC, F1 and recall scores. Classifier was then refitted on the training dataset with the parameters that resulted in best cross-validated AUC score. Final classification performance was evaluated using accuracy, sensitivity, specificity, F1 and AUC scores.

**Figure 2:**
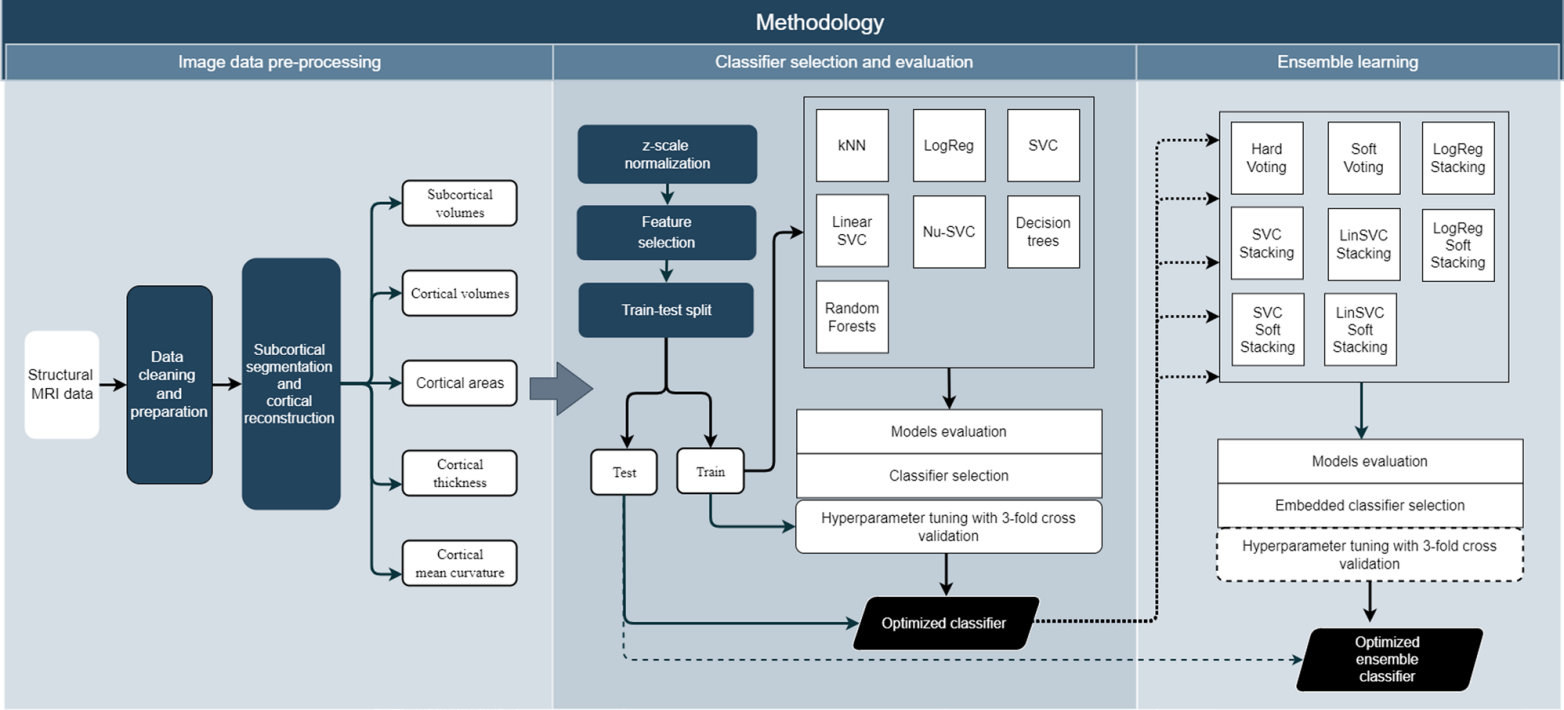
Overview of classification methodology.

For Ensemble classification, input classifiers trained on measure subsets were fused using voting or stacking classifiers. Ensemble classification was done in a manner similar to that of initial base classifier, with initial classifier chosen from a total of 8 Ensemble classifiers. Baseline performance of hard voting, soft voting, hard stacking, and soft stacking, using three different estimators—logistic regression, SVC, Linear SVC and Nu-SVC was tested before choosing initial Ensemble classifier. If stacking classifiers were chosen for Ensemble, they were further tuned for best performance using *GridSearchCV* and threefold cross-validation. Overview of the methodology is seen in Fig. 2.

### Correlation of QoL with neuroimaging features

To evaluate relationship between QoL and key neuroimaging features in schizophrenia cohort, we correlated ΔQoL with ΔFeatures, where ΔQoL is difference between QoL for a patient to mean value for healthy control cohort (QoL − meanQoL_HC_) and ΔFeatures is the difference between neuroimaging feature value for patient and mean value of the same feature for the healthy control cohort (Feature_patient_ − meanFeature_HC_). This ΔQoL and ΔFeatures correlation testing was carried out using Spearman's rank-order correlation and was corrected for multiple comparisons using Bonferroni method.

### Study design

A total of 8 analyses were carried out using Subcortical Volumes (SV), Cortical Volumes (CV), Cortical Areas (CA), Cortical Thickness (CT) and Cortical Mean Curvature (CMC) as feature sets for classification of schizophrenia and healthy controls. An overview of these analyses with feature subsets used, total number of available features and number of selected features is given in Table 1.

**Table 1:** Overview of analyses performed—measures, number of available features and selected number of features used.

Study	Measures used	Total number of available features	Threshold value for feature selection	Number of selected features
1	Subcortical volumes (SV)	55	0.283	23
2	Cortical volumes (CV)	70	0.266	28
3	Cortical areas (CA)	72	0.294	26
4	Cortical thickness (CT)	72	0.389	36
5	Cortical mean curvature (CMC)	70	0.329	28
6	All measures (SV + CV + CA + CT + CMC)	331	0.074	139
7	Ensemble with 3 inputs (SV, CV, CA + CT + CMC)	(a) SV—55 (b) CV—70 (c) CA + CT + CMC—210	–	(a) SV—23 (b) CV—28 (c) CA + CT + CMC—85
8	Ensemble with 5 inputs (SV, CV, CA, CT, CMC)	(a) SV—55 (b) CV—70 (c) CA—72 (d) CT—72 (e) CMC—70	-	(a) SV—23 (b) CV—28 (c) CA—26 (d) CT—36 (e) CMC—28

#### Classification using independent measures

The first set of analyses, analyses 1–5 of Table 1, were conducted using an independent feature set for their classification performance. Classifiers were trained on individual subcortical or cortical measures and optimized as described in “Machine learning based classification”. Additionally, for this set of analyses, correlation of selected features with QoL has been carried out, as given in “Correlation of QoL with neuroimaging features”.

#### Classification using all measures

In the second set of 3 analyses, analyses 6–8 of Table 1, all subcortical and cortical measures were used, either by merging feature sets or through Ensemble methods. For direct comparison with Ensemble methods, we carried out analysis 6 of Table 1, where all subcortical and cortical measures were merged, redundant variables were removed and feature selection and classification were carried out in a manner similar to independent measures classification. In analysis 7, we employed three different input classifiers for Ensemble classification, where input classifiers were trained on subcortical volumes, cortical volumes and remaining cortical measures respectively as shown in Table 1. In analysis 8, five different input classifiers trained on independent measure sets, from analyses 1–5, are used for Ensemble classification.

## Results

**Figure 3:**
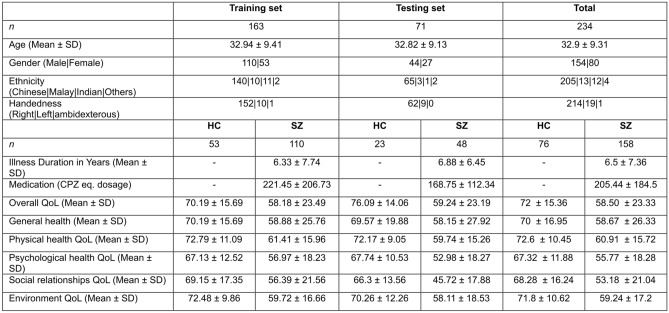
Demographics of patients and controls in training and test datasets.

In our dataset of 234 patients and healthy controls, training set consisted of 163 samples (53 HC, 110 SZ) and testing set consisted of 71 samples (23 HC, 48 SZ). Demographics of patients and controls in this training and test datasets, with group QoL item scores is given in Fig. 3.

### Classification using independent measures and QoL correlation

**Figure 4:**
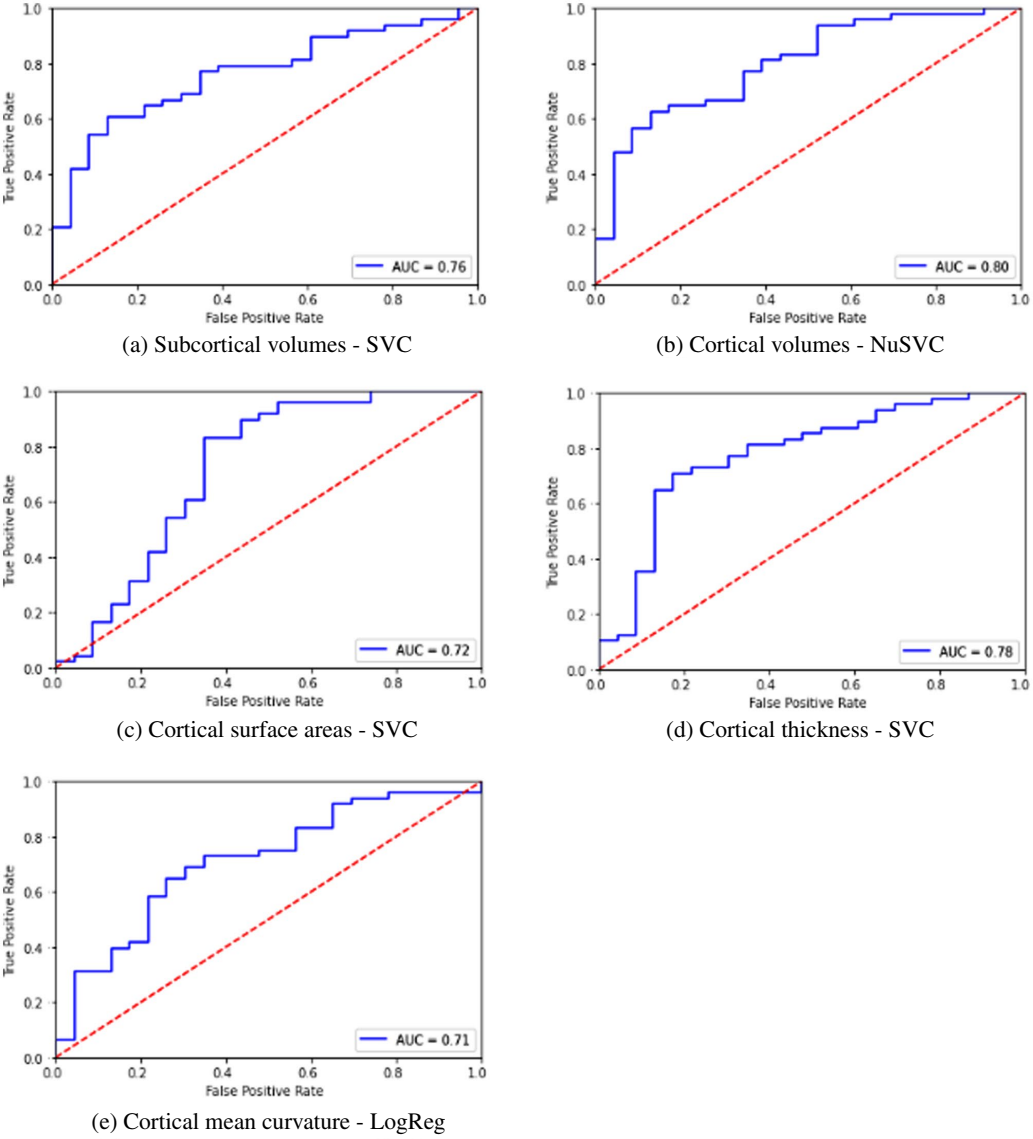
ROC plots for classification using independent feature sets.

When independent measure sets like SV, CA, CV etc. were used, classification accuracy and sensitivity were above 70% and F1 score greater than 0.70 was achieved. Specificity in general was lower between 55 and 65%. Of all independent measures tested, using cortical thickness measures resulted in higher classification accuracy, sensitivity and comparable specificity and F1 scores to other neuroanatomical measures. Among the classifiers, SVM based classifiers and Logistic regression classifiers gave the highest classification performance compared to other classifiers. Results of independent measures-based classification is shown in Table 2, with corresponding ROC curves shown in Fig. 4 from (a) to (e). Key neuroanatomical features selected are given in Supplementary Data A.1–A.5.

**Table 2:** Classification performance using independent measures.

Study	Measures used	Classifier	Parameters tuned	Accuracy	Sensitivity	Specificity	F1	AUC
1	Subcortical volumes	Support Vector Classifier	Regularization parameter C, Max iterations	72%	79%	57%	0.79	0.68
2	Cortical volumes	Nu-Support Vector Classifier	Regularization parameter C, Max iterations, Kernel	73%	81%	57%	0.73	0.69
3	Cortical surface areas	Support Vector Classifier	Regularization parameter C, Max iterations	73%	77%	65%	0.74	0.72
4	Cortical thickness	Support Vector Classifier	Regularization parameter C, Max iterations	75%	81%	61%	0.75	0.71
5	Cortical mean curvature	Logistic Regression	Regularization parameter C, Max iterations, penalty, solver	70%	73%	65%	0.71	0.69

**Figure 5:**
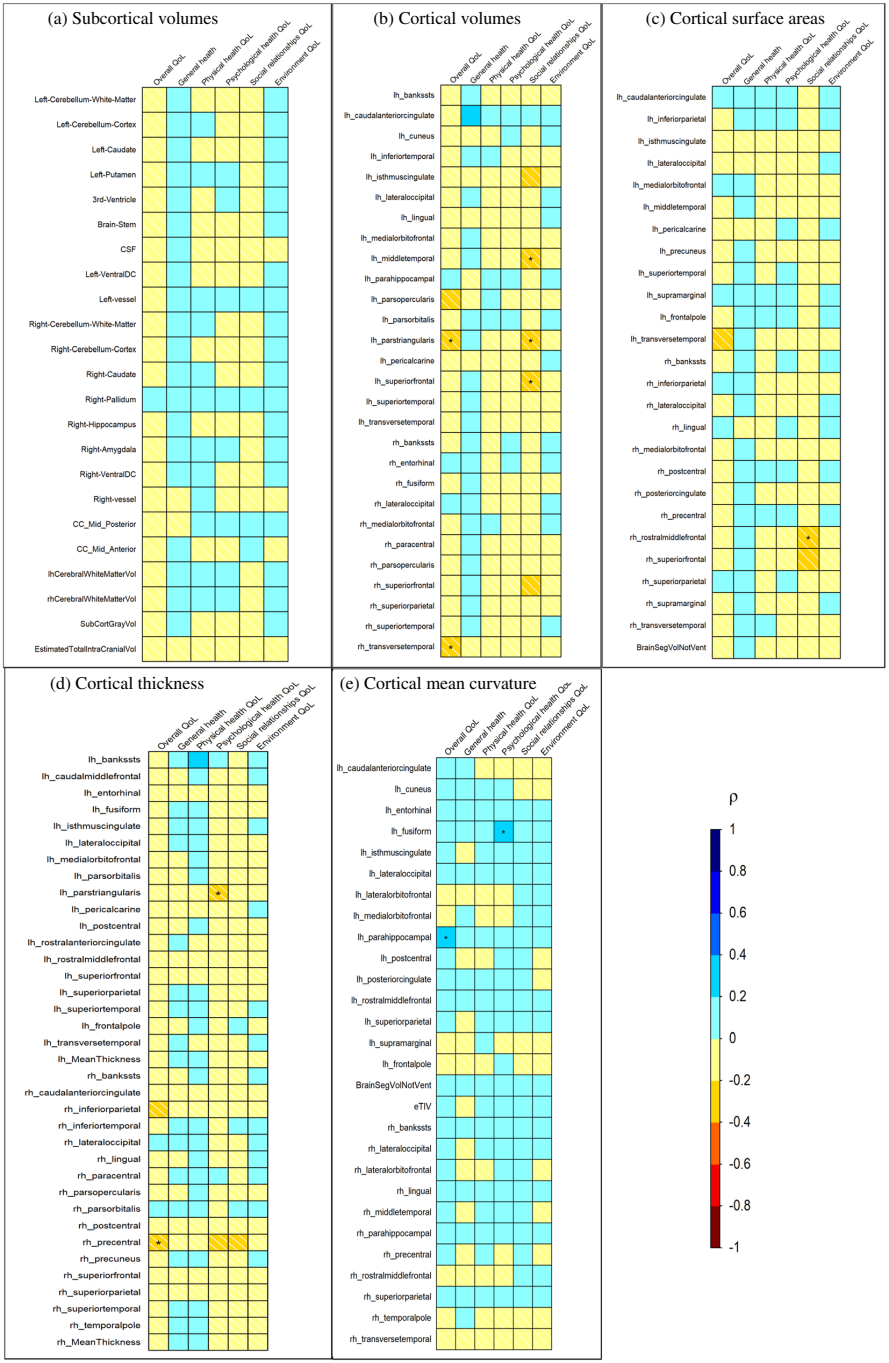
Spearman's rank-order correlation of the ΔQoL items and ΔFeatures, with feature sets from studies 1–5 i.e., (**a**) subcortical volumes, (**b**) cortical volumes, (**c**) cortical surface areas, (**d**) cortical thickness and (**e**) cortical mean curvature (*p-value < 0.05).

Figure 5 shows the Spearman’s correlation between ΔQoL items and ΔFeatures from analyses 1–5, color-coded by Spearman's rank correlation coefficient value, ρ, and significance level highlighted in asterisks. A weak to moderate correlation was found between ΔQoL and ΔFeatures, with significant correlation (p < 0.05) for several features ranging from a ρ of ± 0.2 to ± 0.4. As shown in Fig. 5a, there was no significant correlation between any of the subcortical volume features and ΔQoL. Several cortical volume features were identified to have significant correlations with ΔQoL, as shown in Fig. 5b. Negative correlations were found between left pars triangularis volume and right transverse temporal volume with overall QoL and left pars triangularis, left middle temporal and superior frontal volumes with social relationships domain. Figure 5c shows ΔQoL correlations with cortical surface features, where only the right rostral middle frontal surface area was negatively correlated with the social relationships domain. In cortical thickness measures shown in Fig. 5d, the left pars triangularis region negatively correlated with the psychological health domain of QoL while the right precentral region negatively correlated with overall QoL. Two positive correlations were found with cortical mean curvature features, between left fusiform mean curvature and psychological health domain and left parahippocampal curvature with overall QoL, as shown in Fig. 5e.

### Classification using all measures

**Figure 6:**
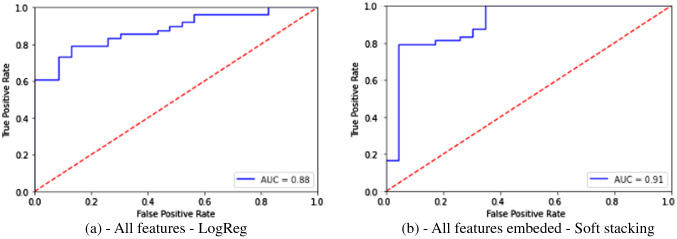
ROC plots for classification using all feature sets.

For analyses 6–8, where all neuroanatomical measures were used either by prior merging or through Ensemble classification, an increase in classification performance was observed. Compared to direct classification using merged features, Ensemble classification resulted in increased accuracy and sensitivity, although decrease in specificity was observed. Hard voting using three input classifiers—SVM trained on subcortical volumes, Nu-SVM trained on cortical volumes and logistic regression classifier trained on remaining cortical measures (areas, thickness and mean curvature) gave an accuracy of 87%, sensitivity of 90% and specificity of 70%. However, when Ensemble classification was carried out using five input classifiers from analyses 1–5 where each was trained on independent features sets, accuracy and sensitivity increased to 87% and 98% but specificity reduced to 65%. Results from classification for these analyses are given in Table 3 below with ROC plots for analyses 6 and 8 are given in Fig. 6.

**Table 3:** Classification performance using all measures.

Study and description	Classifier(s)	Parameters tuned	Accuracy	Sensitivity	Specificity	F1	AUC
6—All subcortical and cortical measures	Logistic Regression	Regularization parameter C, Max iterations, penalty, solver	77%	79%	74%	0.83	0.77
7—Ensemble with 3 inputs (a) SV (b) CV (c) CA + CT + CMC	Ensemble—Hard Voting (a) Support Vector Classifier (b) Nu-Support Vector Classifier (c) Logistic Regression		83%	90%	70%	0.83	0.80
8—Ensemble with 5 inputs (a) SV (b) CV (c) CA (d) CT € CMC	Ensemble—Soft Stacking (a) Support Vector Classifier (b) Nu-Support Vector Classifier (c) Support Vector Classifier (d) Support Vector Classifier (e) Logistic Regression	Regularization parameter C, Max iterations, penalty, solver	87%	98%	65%	0.87	0.82

## Discussion and conclusion

In this study, we investigated classification of subjects with schizophrenia and healthy controls using diverse neuroanatomical measures, including subcortical and cortical structure volumes, cortical surface areas, mean curvature and thickness of cortical structures. Some of these neuroanatomical measures have not been studied so far and hence their role and utility in classifying the two cohorts is unclear. Hence our work on classification using these independent feature sets, provides a baseline for future studies in this direction. From our results, classification performance was comparable between independent measure sets, with accuracy, sensitivity and specificity ranging from 70–73%, 73–81% and 57–61% respectively. Among all the measures, employing cortical thickness as the feature set resulted in slightly higher accuracy and sensitivity. In these single measure set based classification, SVM-based classifiers and logistic regression classifiers consistently gave better classification performances compared to other tested classifiers for measures, which was also reported by Yassin et al.^16^ Additionally, we also evaluated classification performance using Ensemble classification using all available measures. Employing a diverse set of measures however resulted in much improved accuracy, sensitivity and specificity, with ranges of 77–87%, 79–98% and 65–74% respectively. In Ensemble classification with 5 different input classifiers, one from each measure set, and 141 of all available 339 neuroanatomical features were employed. This resulted in highest accuracy and sensitivity of 87% and 98% respectively. These 141 features were further correlated with QoL scores of patients with schizophrenia which revealed a weak to moderate correlation. With overall QoL, volumes of left pars triangularis and right transverse temporal regions, thickness of right precentral region were negatively correlated while mean curvature of left parahippocampal region was positively correlated. With the psychological health domain of QoL, thickness of left pars triangularis was negatively correlated and mean curvature of left fusiform was positively correlated. Volumes of left pars triangularis, left middle temporal and superior frontal regions, and surface area of right rostral middle frontal region negatively correlated with social relationship domain of QoL.

Compared to single measure based classification models from our own analyses as well as from literature, we observed that employing multiple measures increased classification performance. Specifically, using Ensemble methods resulted in much higher accuracy and sensitivity compared to direct classification from measures. In Ensemble classification, a new classifier is generated with inputs from various base classifiers. Such a learning process performed better than any single input classifier and allowed for increased classification performance, reducing bias and variance. We attributed improved performance of our classification to usage of Ensemble methods as well as utilization of multiple neuroanatomical measures for classification. Although sensitivity–specificity trade-off was observed as we increased the number of input classifiers, specificity achieved was still comparable to those obtained from single measure classification analyses within this study. Further studies in this direction on different datasets could employ hybrid and ensemble machine learning or deep learning methods which have been shown to improve classification performance^44^.

**Figure 7:**
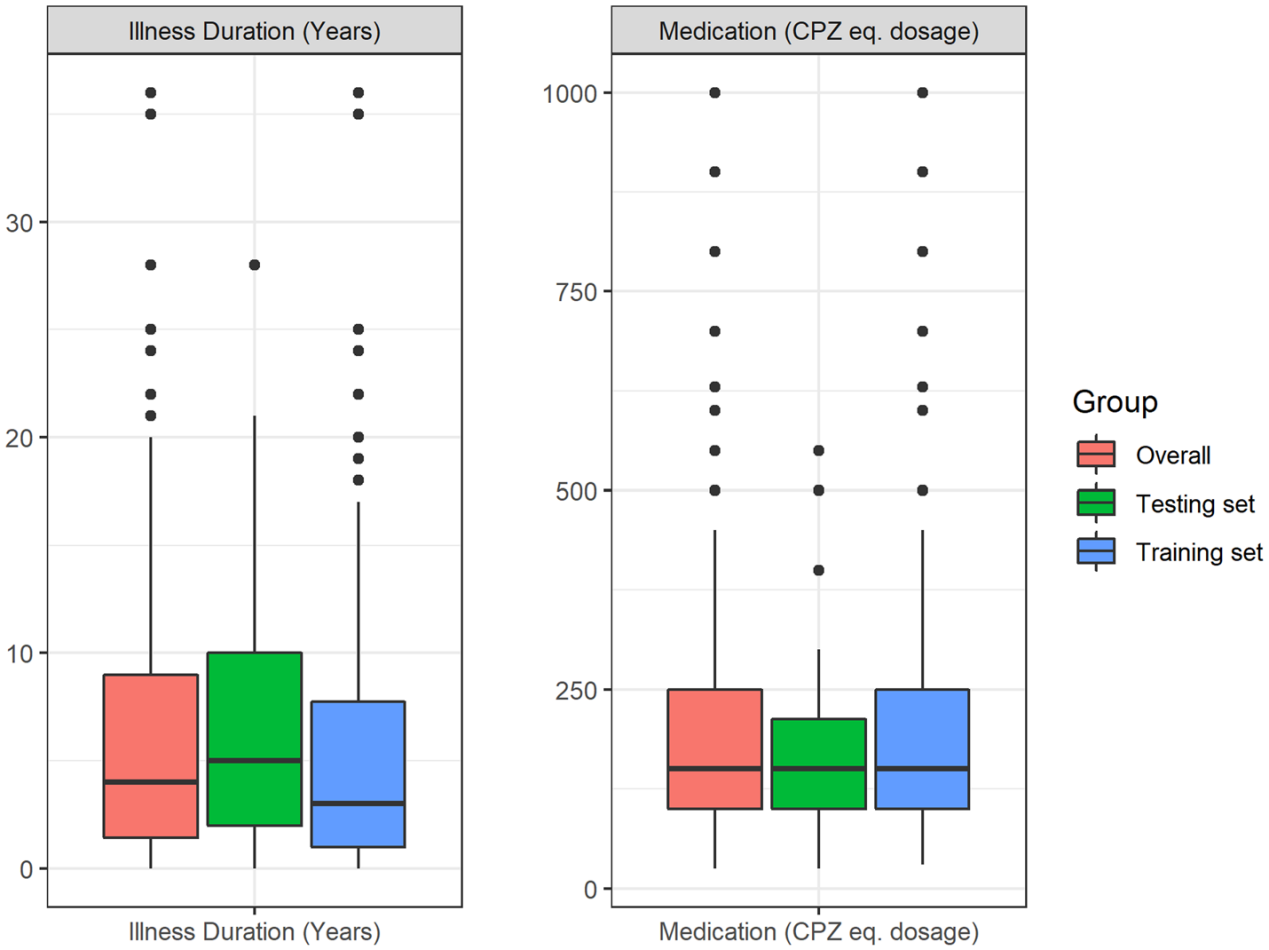
Illness duration (in years) and medication status (chlorpromazine equivalent dosage) distribution in patient dataset.

In our study, we employed neuroanatomical features from both the left and right hemisphere separately for feature selection. Among these measures, certain regions have been identified from feature selection to be important in both the hemispheres. Pericalcarine region seemed to play a key role in Ensemble classification, with its surface area, cortical mean curvature and mean thickness measures selected and employed. This was followed by volume and thickness features of medial orbitofrontal and superior temporal regions, volume and surface area features of transverse temporal region and mean curvature and thickness features of rostral middle frontal regions. Among other features that contributed to classification, in both left and right hemispheres are volumes of pars opercularis, superior frontal, lateral occipital, banks of superior temporal sulcus, surface areas of isthmus of cingulate and supramarginal region, curvature of caudal anterior cingulate, precentral, superior parietal, parahippocampal, temporal pole regions and insula and thickness of pars orbitalis, inferior parietal and postcentral regions. Among subcortical measures, volumes of putamen in left hemisphere, amygdala, hippocampus and pallidum in right hemisphere, caudate, mid anterior and mid posterior regions of corpus callosum, cerebral white matter volume, ventral diencephalon and subcortical gray volume were identified as important features. Volumes of brain stem, cerebellum cortex, cerebellum white matter, brain segmentation, CSF, right and left vessel, 3rd Ventricle and total intracranial volume were also among key selected features for classification. However, it is important to note that our dataset consists of patients with varying illness and medication status, as shown in Fig. 7. When we further analysed the Ensemble results after excluding 13 subjects receiving > 500 CPZ eq mg/day within our modest sample, we found that whilst there is a mild decrease in accuracy (87 to 81%), sensitivity (98 to 89%) and AUC (0.82 to 0.77) for Ensemble with 5 inputs, the specificity and F1 remained the same. The overall pattern of gains in employing the Ensemble methods specifically Ensemble with 5 inputs remained. Additional studies using a larger and more normally distributed dataset can evaluate the utility and role of neuroanatomical markers at each stage of illness and treatment.

There have been very few studies which examined the correlations between neuroanatomical measures and quality of life assessments. For instance, our findings of negative correlation between social relationship subscale of QoL and cortical volumes (temporal, frontal regions) were consistent with those within a study by Ubukata et al.^45^ Using voxel-based morphometry and Japanese version of the Schizophrenia Quality of Life Scale (JSQLS), they found that the psychosocial subscale QoL score is negatively correlated with gray matter volume in bilateral middle frontal gyrus, left midbrain, left postcentral gyrus, left inferior temporal gyrus, left inferior frontal gyrus, right middle occipital gyrus, and right cerebellum. Motivation/energy subscale QoL score was found to be negatively correlated with gray matter volume in the left superior frontal sulcus, left parahippocampal gyrus, left inferior temporal gyrus, right fusiform gyrus, right amygdala, right lingual gyrus, bilateral middle frontal gyrus, right superior temporal gyrus, right postcentral gyrus, and left middle temporal gyrus. Of note, the clinical factors subscale score was negatively correlated with GM volume in the left inferior frontal gyrus, left precentral gyrus, right middle frontal gyrus, left fusiform gyrus, and left inferior temporal gyrus. Another study^46^ which carried out correlation of features with objective Quality of Life Scale (QLS) reported that instrumental role category score from the four subscales was correlated with the right anterior insula.

Several limitations are to be noted. First, we tested this classification system on a modest cross sectional data set. Second, further efforts to assess the utility of this classification system within a longitudinal dataset would allow better understanding and optimization of the classifiers over the time-course of illness. Third, we did not examine the use of the classifiers in differentiating subtypes of the illness by specific psychopathology, functional course or treatment response. Future studies applying and extending the current parameter-tuning may want to focus on parcellating heterogeneity of illness pertaining to specific symptomatology such as hallucinations, delusions, first rank symptoms using classification systems or even incorporating other clinical modalities such as cognitive functioning, treatment variables and functional factors within such classifiers in a larger dataset drawn from different and larger cohorts of subjects.

## Supplementary Information

Supplementary Information.
