# Supplementary material for: Machine learning classification of schizophrenia patients and healthy controls using diverse neuroanatomical markers and Ensemble methods
Source: Sci Rep. 2022 Feb 17;12:2755. doi: 10.1038/s41598-022-06651-4 (PMC8854385; doi:10.1038/s41598-022-06651-4)
Supplement: Supplementary file 1 — Supplementary Information. [file 41598_2022_6651_MOESM1_ESM.docx]

Supplementary Information

1. Regions contributing to discrimination of patients with schizophrenia and healthy cohorts
   1. Selected volume features from segmentation

Left Putamen, Right Pallidum, Right Hippocampus, Right Amygdala, Corpus Callosum_Mid_Posterior, Corpus Callosum_Mid_Anterior, 3rd Ventricle, Brain Stem, CSF, Caudate, Cerebellum White Matter, Cerebellum Cortex, VentralDC, Right and Left vessel, Cerebral White Matter Volume, SubCortical Gray Volume, Estimated total intracranial volume.

- 1. Selected volume features from cortical parcellation

| **Selected cortical volume features** | **Hemisphere** | **Lobe** |
| --- | --- | --- |
| caudalanteriorcingulate | lh | Frontal |
| medialorbitofrontal | lh | Frontal |
| medialorbitofrontal | rh | Frontal |
| paracentral | rh | Frontal |
| parsopercularis | lh | Frontal |
| parsopercularis | rh | Frontal |
| parsorbitalis | lh | Frontal |
| parstriangularis | lh | Frontal |
| superiorfrontal | lh | Frontal |
| superiorfrontal | rh | Frontal |
| cuneus | lh | Occipital |
| lateraloccipital | lh | Occipital |
| lateraloccipital | rh | Occipital |
| lingual | lh | Occipital |
| pericalcarine | lh | Occipital |
| isthmuscingulate | lh | Parietal |
| superiorparietal | rh | Parietal |
| bankssts | lh | Temporal |
| bankssts | rh | Temporal |
| entorhinal | rh | Temporal |
| fusiform | rh | Temporal |
| inferiortemporal | lh | Temporal |
| middletemporal | lh | Temporal |
| parahippocampal | lh | Temporal |
| superiortemporal | lh | Temporal |
| superiortemporal | rh | Temporal |
| transversetemporal | lh | Temporal |
| transversetemporal | rh | Temporal |
|  |  |  |

- 1. Selected surface area features from cortical parcellation

| **Selected cortical surface area features** | **Hemisphere** | **Lobe** |
| --- | --- | --- |
| caudalanteriorcingulate | lh | Frontal |
| caudalmiddlefrontal | rh | Frontal |
| frontalpole | lh | Frontal |
| medialorbitofrontal | lh | Frontal |
| paracentral | rh | Frontal |
| parsopercularis | rh | Frontal |
| parsorbitalis | lh | Frontal |
| precentral | rh | Frontal |
| lateraloccipital | lh | Occipital |
| pericalcarine | lh | Occipital |
| pericalcarine | rh | Occipital |
| inferiorparietal | lh | Parietal |
| isthmuscingulate | lh | Parietal |
| isthmuscingulate | rh | Parietal |
| postcentral | lh | Parietal |
| supramarginal | lh | Parietal |
| supramarginal | rh | Parietal |
| bankssts | lh | Temporal |
| inferiortemporal | lh | Temporal |
| middletemporal | rh | Temporal |
| parahippocampal | rh | Temporal |
| temporalpole | lh | Temporal |
| transversetemporal | lh | Temporal |
| transversetemporal | rh | Temporal |
| BrainSegVolNotVent |  |  |
| Estimated total intracranial volume |  |  |
|  |  |  |

- 1. Selected mean curvature features from cortical parcellation

| **Selected cortical mean curvature features** | **Hemisphere** | **Lobe** |
| --- | --- | --- |
| caudalanteriorcingulate | lh | Frontal |
| caudalanteriorcingulate | rh | Frontal |
| caudalmiddlefrontal | rh | Frontal |
| frontalpole | lh | Frontal |
| parsorbitalis | lh | Frontal |
| precentral | lh | Frontal |
| precentral | rh | Frontal |
| rostralanteriorcingulate | lh | Frontal |
| rostralmiddlefrontal | lh | Frontal |
| rostralmiddlefrontal | rh | Frontal |
| superiorfrontal | lh | Frontal |
| lateraloccipital | lh | Occipital |
| lingual | lh | Occipital |
| pericalcarine | lh | Occipital |
| pericalcarine | rh | Occipital |
| isthmuscingulate | lh | Parietal |
| postcentral | lh | Parietal |
| precuneus | rh | Parietal |
| superiorparietal | lh | Parietal |
| superiorparietal | rh | Parietal |
| supramarginal | lh | Parietal |
| inferiortemporal | rh | Temporal |
| middletemporal | lh | Temporal |
| parahippocampal | lh | Temporal |
| parahippocampal | rh | Temporal |
| superiortemporal | rh | Temporal |
| temporalpole | lh | Temporal |
| temporalpole | rh | Temporal |
| insula | - |  |
|  |  |  |

- 1. Selected thickness features from cortical parcellation

| **Selected cortical mean thickness features** | **Hemisphere** | **Lobe** |
| --- | --- | --- |
| caudalanteriorcingulate | rh | Frontal |
| caudalmiddlefrontal | lh | Frontal |
| medialorbitofrontal | lh | Frontal |
| medialorbitofrontal | rh | Frontal |
| paracentral | rh | Frontal |
| parsorbitalis | lh | Frontal |
| parsorbitalis | rh | Frontal |
| rostralanteriorcingulate | lh | Frontal |
| rostralmiddlefrontal | lh | Frontal |
| rostralmiddlefrontal | rh | Frontal |
| superiorfrontal | rh | Frontal |
| cuneus | lh | Occipital |
| lateraloccipital | rh | Occipital |
| lingual | lh | Occipital |
| pericalcarine | lh | Occipital |
| pericalcarine | rh | Occipital |
| inferiorparietal | lh | Parietal |
| inferiorparietal | rh | Parietal |
| isthmuscingulate | lh | Parietal |
| postcentral | lh | Parietal |
| postcentral | rh | Parietal |
| precuneus | rh | Parietal |
| superiorparietal | rh | Parietal |
| bankssts | lh | Temporal |
| entorhinal | lh | Temporal |
| inferiortemporal | rh | Temporal |
| middletemporal | rh | Temporal |
| superiortemporal | lh | Temporal |
| superiortemporal | rh | Temporal |
| temporalpole | lh | Temporal |
|  |  |  |
